# ΔM4: Membrane-Active Peptide with Antitumoral Potential against Human Skin Cancer Cells

**DOI:** 10.3390/membranes13070671

**Published:** 2023-07-14

**Authors:** Estefanía Fandiño-Devia, Gloria A. Santa-González, Maria C. Klaiss-Luna, Ibeth Guevara-Lora, Verónica Tamayo, Marcela Manrique-Moreno

**Affiliations:** 1 Chemistry Institute, Faculty of Exact and Natural Sciences, University of Antioquia, A.A. 1226, Medellin 050010, Colombia; liliana.fandino@udea.edu.co (E.F.-D.); maria.klaiss@udea.edu.co (M.C.K.-L.); veronica.tamayo@udea.edu.co (V.T.); 2 Grupo de Investigación e Innovación Biomédica, Facultad de Ciencias Exactas y Aplicadas, Instituto Tecnológico Metropolitano, A.A. 54959, Medellín 050010, Colombia; gloriasanta@itm.edu.co; 3 Department of Analytical Biochemistry, Faculty of Biochemistry, Biophysics and Biotechnology, Jagiellonian University, 30-387 Krakow, Poland; ibeth.guevara-lora@uj.edu.pl

**Keywords:** membrane-acting peptides, antitumoral peptides, skin cancer, melanoma, epidermoid carcinoma, membrane–peptide interactions

## Abstract

Peptides have become attractive potential agents due to their affinity to cancer cells. In this work, the biological activity of the peptide ΔM4 against melanoma cancer cell line A375, epidermoid carcinoma cell line A431, and non-tumoral HaCaT cells was evaluated. The cytotoxic MTT assay demonstrates that ΔM4 show five times more activity against cancer than non-cancer cells. The potential membrane effect of ΔM4 was evaluated through lactate dehydrogenase release and Sytox uptake experiments. The results show a higher membrane activity of ΔM4 against A431 in comparison with the A375 cell line at a level of 12.5 µM. The Sytox experiments show that ΔM4 has a direct effect on the permeability of cancer cells in comparison with control cells. Infrared spectroscopy was used to study the affinity of the peptide to membranes resembling the composition of tumoral and non-tumoral cells. The results show that ΔM4 induces a fluidization effect on the tumoral lipid system over 5% molar concentration. Finally, to determine the appearance of phosphatidylserine on the surface of the cell, flow cytometry analyses were performed employing an annexin V–PE conjugate. The results suggest that 12.5 µM of ΔM4 induces phosphatidylserine translocation in A375 and A431 cancer cells. The findings of this study support the potential of ΔM4 as a selective agent for targeting cancer cells. Its mechanism of action demonstrated selectivity, membrane-disrupting effects, and induction of phosphatidylserine translocation.

## 1. Introduction

Skin cancer is one of the most common types of malignancies in humans, and is caused by the out-of-control growth of abnormal cells in the epidermis [1]. General risk factors for this pathology include prolonged exposure of the skin to ultraviolet (UV) radiation, sunlight and tanning beds, the thinning of the ozone layer, epidermic lesions such as moles, genetic factors, and a compromised immune system [2]. Epidemiological surveillance indicates a continuous increase in the number of cases every year, which translates into significantly higher costs to public health services [3,4]. Around 2–3 million new cases are reported worldwide per year and the incidence rates are predicted to be higher in the coming decades [5].

The main types of skin cancer are basal cell carcinoma (BCC), squamous cell carcinoma (SCC), and melanoma. The last of these accounts for less than 5% of skin cancers but is responsible for 80% of skin-cancer-related deaths [6,7]. It is the deadliest form of skin cancer because it is much more likely to spread to other parts of the body (metastasize) if it is not removed or treated in the first stages. The selected treatment depends on several factors, such as the progression of the disease, and the size and location of the malignancy. Early-detected melanomas are mainly treated by surgical excision, which can result in negative consequences such as concerns related to appearance; social implications, including difficulty in social activities and interactions; and adverse effects such as pain and swelling, leading to emotionally and physically adverse consequences for patients [8,9]. Radiotherapy is not used as a first-line treatment; it is used only in cases where surgery is not the primary option [10]. Additionally, if the tumors are large and the characteristics of the malignancy make the patient a poor surgical candidate, alternative therapies are available, such as topical therapy [11]. This is commonly recommended for patients presenting with multiple clinical and subclinical lesions over a large anatomical area. Treatment with topical agents allows higher drug levels to be reached at the site of the tumor, with potentially less toxicity to the patient than systemic agents. Among the most frequently used topical agents are 5-fluorouracil, used for the treatment of BCC [12]; and imiquimod, which was approved in 2004 for treatment of actinic keratosis and superficial BCC in patients where surgery or alternative treatments were not suitable [13]. In advanced or metastatic cases of melanoma, immunotherapy is used with the administration of agents such as ipilimumab, nivolumab, and pembrolizumab. In this treatment, the immune system is activated to detect and destroy the cancer cells [14,15]. Unfortunately, it is not an innocuous therapy, and several adverse effects including abdominal pain, severe diarrhea, blood in stool, life-threatening skin rash, nephritis, pneumonitis, pancreatitis, and non-infectious myocarditis have been reported [14,16].

A general characteristic of the most extensively used chemotherapeutics is that they cannot differentiate between cancer and non-cancer cells, and being responsible for the side effects of these drugs. Due to this reason, it is necessary to design and evaluate novel molecules for the potential treatment of cancer. Cationic active peptides (CAPs) are part of the natural repertory of molecules that constitute the innate immune system of several organisms and participate in the first line of defense in response to the attack of pathogens. These molecules are composed of 50 residues or fewer, the majority of which have a net positive charge and are amphipathic [17,18]. Thousands of natural and synthetic peptides have been reported, which have a broad spectrum of activity, including antitumoral activity in several cancer cell lines. They are attractive potential agents, because they execute their biological activity via a non-specific mechanism, which means cells are less likely to develop resistance [19].

The mechanism of action of CAPs is based on electrostatic interactions between the cationic residues on the peptide and anionic groups of the target membrane, such as phosphatidylserine (PS), sialic acid, or heparin sulfate [20]. Based on this interaction, cancer cells are more susceptible to interact with the peptides, in comparison with non-cancer cells. It has been demonstrated that short host defense-like peptides exert their mechanism of action by binding the PS exposed in the outer leaflet of cancer cells [21,22]. Varontsova et al. reported the cytotoxic activity of the Latarcins 2a, a peptide extracted from the *Lachesana tarabaevi* spider venom, against human erythroleukemia K562 cells. The authors demonstrate that the peptide binds to the membrane surface of K562 cells, triggering the PS externalization [23]. Another case of selective PS destabilization mechanism was published by Herrera-León and co-workers. They demonstrate by different biophysical and in silico approaches that the synthetic peptide HB43, active against several cancer cell lines, preferentially interacts with negatively charged vesicles in comparison with the zwitterionic ones. The authors probed by MD the formation of specific interactions between the lysine side chains and the carboxylate group of phosphatidylserine of membrane lipids [24]. Wang and co-workers reported that the peptide temporin-1CEa exerted preferential cytotoxic activity toward cancer cells. They demonstrate that the negatively charged PS expressed on A375 melanoma cancer cell surface acts as a target for temporin-1CEa [25].

Previous studies by our research group focused on the effects of the synthetic peptide ΔM4 on the mitochondrial function and cell cycle progression of melanoma cells [26]. In the present study, we further investigated the viability effect of the synthetic peptide ΔM4 on human melanoma cells (A375), epidermoid carcinoma cells (A431), and spontaneously immortalized human keratinocytes (HaCaT) by MTT method. Additionally, to monitor the interaction of ΔM4 with cell membranes, lactate dehydrogenase release assay and flow cytometry were applied. Biophysical studies on model membranes give valuable insights into peptides’ mechanism of action. For this reason, this work also presents the biophysical evaluation by infrared spectroscopy of the interaction of the synthetic peptide ΔM4 and a multicomponent lipid system representative of cancer cell membranes. These experiments were designed to explore the affinity of ΔM4 for the lipid membranes, and to follow the conformational change of the peptide in the presence of the cell membranes. Finally, flow cytometry experiments to determine the potential exposure of phosphatidylserine, which is indicative of apoptosis, were performed.

## 2. Materials and Methods

### 2.1. Reagents

ΔM4 (NFFKRIRRAWKRIWKWIYSA, Lot. b88771380001/PE2074) and LTX-315 (KKWWKKWDipK–NH_2_, Lot. 91799130007/PE4961) were purchased through a custom peptide synthesis service from GenScript (Piscataway Township, NJ, USA). The solvent removal was performed, the peptide purity was determined by analytical HPLC (higher than 95%), and the molecular weight was confirmed with MALDI-TOF mass spectrometry. Dipalmitoylphosphatidylcholine (DPPC, Lot. 160PC-318), sphingomyelin egg chicken (SM, Lot. 860061P-25MG-A-116), dipalmitoylphosphatidylethanolamine (DPPE, Lot. 160PE-106), dipalmitoylphosphatidylserine sodium salt (DPPS, Lot. 840037P-500MG-A-078CL750332P-200MG-A-030), palmitoyloleoylphosphatidylcholine (POPC, Lot. 850457P-500MG-A-211), palmitoyloleoylphosphatidylethanolamine (POPE, Lot. 850757P-500MG-B-151), and palmitoyloleoylphosphatidylserine sodium salt (POPS, Lot. 840034P-25MG-A-250) were purchased from Avanti Polar Lipids (Alabaster, AL, USA). HEPES, NaCl, and EDTA were purchased from Sigma-Aldrich (St. Louis, MO, USA).

### 2.2. Cell Cultures and Treatments

HaCaT (non-tumoral, CLS 300493, purchased from CLS https://cls.shop/HaCaT/300493, accessed on 21 June 2023), A375 (melanoma, ATCC CRL-1619, purchased from ATCC https://www.atcc.org/products/crl-1619, accessed on 21 June 2023), and A431 (epidermoid carcinoma CRL-1555, purchased from ATCC https://www.atcc.org/products/crl-1555, accessed on 21 June 2023) skin cell lines were cultured in Dulbecco’s modified Eagle’s medium (DMEM) with 5% fetal calf serum (FCS), 100 µg/mL of penicillin, and 100 µg/mL of streptomycin. The cells were maintained at 37 °C in a humidified incubator with a mixture CO_2_/air (5%/95%). Before the experiments, cells were examined under a microscope for correct morphology, adherence, and exponential growth. For experiments, cells were seeded and cultured under standard conditions, after allowing the adhesion of cells, different concentrations of ΔM4 were added to cultures in the presence of complete medium (5% FCS). To evaluate the biological effect, cells were trypsinized, pelleted, and analyzed for different tests. All reported data include at least three independent experiments per treatment group.

### 2.3. Cell Viability Evaluation

A colorimetric 3-(4,5-dimethylthiazol-2-yl)-2,5-diphenyltetrazolium bromide (MTT) viability assay (Sigma, M2128) was employed to evaluate the in vitro cytotoxicity of ΔM4 and LTX-315 against the A375, A431 cancer cells, and the non-malignant HaCaT cells. For the experiments, 1.0 × 10^4^ cells/well were seeded in 96-well plates. After cell adhesion, peptide treatments at 5, 25, 50, and 100 µM were added and incubation was carried out for 24 h. After this period, MTT was added (0.5 mg/mL) and incubation was carried out for 2 h at 37 °C in the dark. Afterwards, 100 µL of isopropyl alcohol (40 mM) was incorporated, and the MTT adduct absorption was measured at 570 nm through a MultiSkan Go (Thermo Fischer Scientific, Waltham, MA, USA). The absolute IC_50_ values were determined using GraphPad Prism software 8.0.1 software by fitting a sigmoidal dose–response curve to the data. This analysis takes into account the minimum and maximum response levels, enabling the calculation of the concentration of the peptide that reduces cell viability by 50%

### 2.4. Interaction between Peptides and Cell Membranes

#### 2.4.1. Release of Lactate Dehydrogenase (LDH)

Enzyme activity was performed using the LDH kit (Takara- MK401) according to the manufacturer’s instructions. 1.0 × 10^4^ cells/well were seeded in 96-well plate, peptide treatments at 5, 12.5, 25, 50 and 100 µM were added and incubation was carried out for 24 h. Next, cell culture supernatants were harvested and quantified for LDH levels at 490 nm through a MultiSkan Go (Thermo Fischer Scientific, Waltham, MA, USA). With the use of triton, 100% cell death was induced.

#### 2.4.2. Permeability Study by Sytox Uptake

Experiments were performed to determine whether ΔM4 could affect the cytoplasmic membrane of A375, A431, and HaCaT cells. For these experiments, 2.5 × 10^5^ cells/well were seeded in 96-well plate, and treated with different concentrations of peptides (12.5, 25 and 50 µM) for 24 h. Afterwards, cells were stained with 1 µL the fluorescent stain Sytox Green^®^ Invitrogen, V35112, component B (Thermo Fischer Scientific, Waltham, MA, USA) at a concentration of 1 µM, and incubated at 37 °C and 5% CO_2_ for 15 min. After the incubation, cells were pelleted and washed three times with PBS. The uptake of the fluorescent stain was quantified by flow cytometry using BD LSRFortessa (BioSciences, Franklin Lakes, NJ, USA) with a 50 mW laser at 488 nm and a flow rate of 66 μL/min. Injection volume was set at 25 μL, and samples were triggered by forward scatter (FSC) allowing for a good separation of cells from background signals. The remaining background noise was electronically excluded from the cell signal even though the threshold was adequately set. For each treatment, a total of 10,000 events were analyzed.

#### 2.4.3. Phase Transition Experiments of Model Membranes by Infrared Spectroscopy

In order to investigate the potential interaction of ΔM4 with the phospholipids present in the non-tumoral and tumoral membranes, experiments with synthetic models were performed according to Li et al. [27] with a small variation, and monitored by infrared spectroscopy. The lipid systems used to mimic non-tumoral and tumoral membrane composition were DPPC/SM/DPPE 4.35:4.35:1 (*w*/*w*) and DPPC/SM/DPPE/DPPS 3.85:3.85:0.8:1.5 (*w*/*w*), respectively. For the tumoral model, a proportion of PS between 10–20% was used, according to Yeung et al. [28]. First, the background was taken using buffer (20 mM HEPES, 500 mM NaCl, and 1 mM EDTA, pH 7.4) in the same temperature range of the sample. The temperature range was set by a Huber Ministat 125 computer-controlled circulating water bath (Huber, Offenburg, Germany) with an accuracy of ±0.1 °C. For the experiments, a BioATR II cell integrated to a Tensor II spectrometer (Bruker Optics, Ettlingen, Germany) with a MCT detector using a spectral resolution of 4 cm^−1^ and 120 scans per spectrum was used. Supported lipid bilayers were formed directly on the BioATR cell. Stock solutions of each lipid system at a concentration of 20 mM were prepared in chloroform to coat the silicon crystal. A total of 20 µL of the lipid stock solution were used to fill the cell. The solvent was evaporated, resulting in a lipid multilayer film. For the measurements, the cell was subsequently filled with 20 µL of buffer or peptide solution and incubated for 10 min over the phase transition temperature of the lipid system.

After the experiment, the position of the ν_s_CH_2_ band was determined by the second derivative method. For this, all the spectra were cut in the 2970–2820 cm^−1^ range, shifted to a zero baseline, and the peak picking function included in the OPUS 7.5 software used. The results of the peak vs. the temperature were plotted. The transition temperature (T_m_) of the lipid systems correspond to the inflection point of the obtained thermal transition curves using the Boltzmann model of the OriginPro 8.0 software (OriginLab Corporation, Northampton, MA, USA).

#### 2.4.4. Secondary Structure Prediction of ΔM4 by Infrared Spectroscopy

A 2 mM solution of ΔM4 was prepared in buffer (10 mM HEPES, 500 mM NaCl, 1 mM EDTA, pH 7.4). Liposomes of the non-tumoral (POPC/SM/POPE 4.35:4.35:1, *w*/*w*) and tumoral (POPC/SM/POPE/POPS 3.85:3.85:0.8:1.5, *w*/*w*) systems [29] were prepared in order to obtain a 6 mM final concentration. The lipids were dissolved in pure chloroform in a glass test tube, the solvent was evaporated, and the solvent traces were removed by a freeze dryer Virtis Benchtop Pro (Sp Scientific, Warminster, PA, USA) for 30 min. Dried lipids were hydrated in buffer, and small unilamellar vesicles (SUVs) were formed by sonicating the samples above the main phase transition temperature of the lipids for at least 15 min. To predict the secondary structure, the peptide was added to the liposome suspension in order to obtain a peptide-to-lipid molar ratio of 15 mol%. The experiments were performed in an AquaSpec cell (Bruker Optics, Ettlingen, Germany) at 37 °C. The secondary structure prediction was achieved using the calibration method supplied by the Confocheck^TM^ system (Bruker Optics, Ettlingen, Germany).

### 2.5. Annexin V Binding Experiments

The annexin V staining method was used to detect potential apoptosis induction by ΔM4 in HaCaT, A375, and A431 cells. Cell lines were treated with 12.5, 25, and 50 µM, cells were pelleted, washed twice with PBS, and diluted to 2.5 × 10^5^ cell/mL. Afterwards, 300 µL of cell suspension with union buffer was added to a flow cytometry tube and stained with 2 µL R-phycoerythrin (R-PE) annexin (Invitrogen, V35112, component A) at a concentration of 0.25 µg/mL, and maintained at 37 °C for 15 min. The cells were then analyzed for apoptosis by flow cytometry using BD LSRFortessa™ Cell Analyzer (BioSciences, Franklin Lakes, NJ, USA). The data were analyzed using the FlowJo v10.6.2 software with Watson pragmatic model (Tree Star, Ashland, OR, USA).

### 2.6. Statistical Analysis

The results were analyzed using GraphPad Prism 8.0.1 software (GraphPad Software, Boston, MA, USA). The results are expressed as the mean ± standard error of the mean (SEM) of three independent experiments. Analysis of variance (ANOVA) was performed with post hoc comparisons via Fisher’s least significant difference (LSD) test. *p*-values below 0.05 were considered statistically significant. The error bars in the graphs represent the standard errors of the mean (SEMs).

## 3. Results

### 3.1. Viability Effect of ΔM4 on Skin Cells

**Figure 1:**
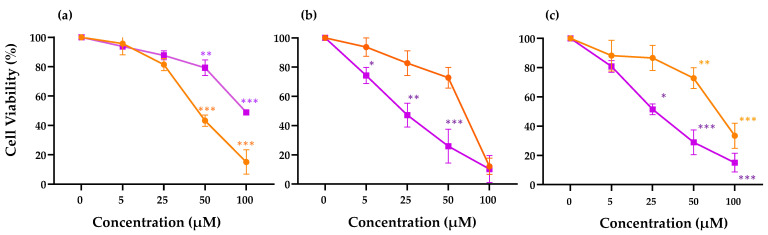
Viability effect of peptides ΔM4 (-■-) and LTX-315 (-●-) at 0, 5, 25, 50, and 100 µM on (**a**) non-tumoral HaCaT cells, (**b**) A375 melanoma cells, and (**c**) A431 carcinoma cells. The differences with respect to non-treated cells were obtained by one-way ANOVA where * *p* ≤ 0.05, ** *p* ≤ 0.01, *** *p* ≤ 0.001.

To determine the in vitro dose-dependent effect of the peptide ΔM4 on the viability of HaCaT epithelial cells, A375 melanoma cells, and A431 carcinoma cells, an MTT assay was performed. The peptide LTX-315, which has been reported to have significant activity in several cancer cell lines including melanoma, was considered as a positive control of the research [30]. The results in Figure 1a show that after 24 h treatment, the peptide only affects the viability of the HaCaT cells at concentrations higher than 50 µM. Meanwhile, the viability results show that at concentrations above 5 µM of ΔM4 (*p* < 0.05), the viability of the A375 and A431 cells are significantly reduced. The results of LTX-315 show a significant reduction in cell viability, but this occurs at a higher concentration in comparison with the concentrations used for ΔM4 (Figure 1b,c).

Table 1 summarizes the determination of the half-maximal inhibitory concentration (IC_50_) of the ΔM4 on the cell lines. The results show that the IC_50_ value obtained is 20 µM for the A375 cells treated with ΔM4, and 26 µM for A431 cells. The effect of the peptide on the viability is significantly lower in HaCaT cells with an IC_50_ of 98 µM. In the case of the control peptide LTX-315, an IC_50_ value of 40 µM is obtained for HaCaT cells, and 65 µM for A431 and A375 cells. Based on the results, the selectivity index (SX) was calculated according to Equation (1). The SX is the ratio that measures the relationship between cytotoxic activity against tumoral and non-tumoral cells. According to the literature, the higher the SX ratio, the more effective and safe a drug should be for potential treatment [31,32]. For a new potential drug, it is expected that the compound would kill cancer cells, but not affect the normal cells. The SX for the peptide ΔM4 is 490 in A375 cells and 377 in A431 cells, values that are indicative of selective activity of the peptide against cancer cells.

(1) $$\mathrm{SX}=\frac{{\;\mathrm{IC}}_{50}\;\mathrm{non}-\mathrm{tumoral}\;\mathrm{cells}}{{\mathrm{IC}}_{50}\;\mathrm{tumoral}\;\mathrm{cells}}\times 100$$

### 3.2. Interaction between Peptides and Cell Membranes

#### 3.2.1. Evaluation of Membrane-Activity of ΔM4 and LTX-315 on Skin Cells

**Figure 2:**
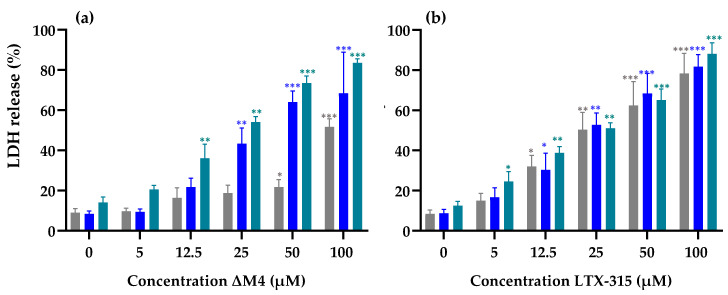
Evaluation of the membrane activity effect of (**a**) ΔM4 and (**b**) LTX-315 against HaCaT (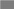), A375 (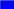), and A431 (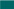) cells. In all cases, cells were treated for 24 h with different concentrations of the peptides (5, 12.5, 25, 50, and 100 µM), and subsequently LDH release was quantified. The bar chart represents the increase in LDH in non-viable cells. The differences with respect to non-treated cells were obtained by one-way ANOVA where * *p* ≤ 0.05, ** *p* ≤ 0.01, *** *p* ≤ 0.001.

To evaluate whether the effect of ΔM4 is associated with the peptide–membrane interactions, the lactate dehydrogenase (LDH) release assay was performed. The results are summarized in Figure 2.

The analysis suggests a concentration-dependent effect of ΔM4 and LTX-315 on the LDH release in cell lines. Figure 2a suggests a higher membrane activity of ΔM4 against A431 in comparison with A375 cell line at concentrations over 12.5 µM. Regarding the effect of ΔM4 against HaCaT cells, the LDH release is lower than 20% at concentrations up to 25 µM. At higher peptide concentrations (50 and 100 µM), the release increases significantly. These results are in accordance with the previous results obtained by MTT assay, where ΔM4 is more active against A375 melanoma and A431 epidermoid carcinoma cell lines in comparison with the HaCaT non-tumoral line. In the case of LTX-315, there is a dose-dependent effect in each cell line, but no differences in the LDH release are detected among the three cell lines at any of the concentrations evaluated (Figure 2b). Due to the fact that the LTX-315 mechanism has been extensively explored [30,33,34,35,36,37,38], and based on the fact that understanding the mechanism of action of a potential new drug is fundamental, the following experiments were performed only for ΔM4.

#### 3.2.2. Changes in Membrane Permeability of ΔM4 on Skin Cells

**Figure 3:**
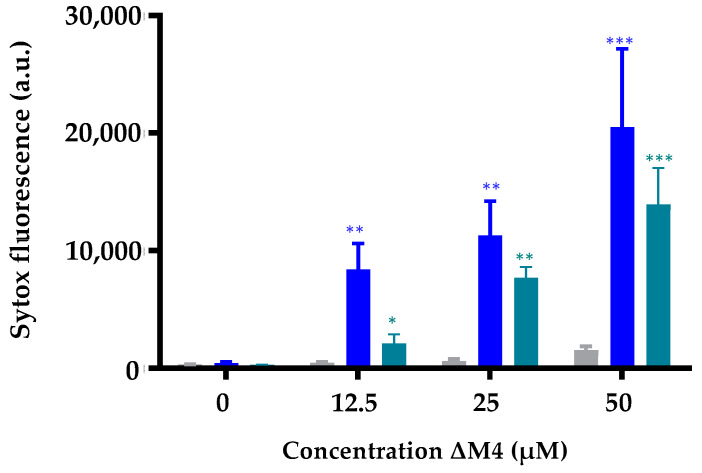
Evaluation of the membrane integrity of HaCaT (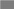), A375 (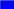), and A431 (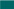) cells after treatment with different concentrations of ΔM4. In all cases, cells were treated for 24 h with different concentrations of ΔM4 (12.5, 25, and 50 µM), and subsequently stained with Sytox Green^®^. The live cells do not uptake colorant, in comparison with the dead cells. The bar chart represents the increase in the fluorescence of Sytox Green^®^ in non-viable cells. The differences with respect to non-treated cells were obtained by one-way ANOVA where the differences for each cell line were found with respect to non-treated cells, where * *p* ≤ 0.05, ** *p* ≤ 0.01, *** *p* ≤ 0.001.

In order to determine the permeability changes induced by the peptide ΔM4 against HaCaT, A375, and A431 cell membranes, intake of the fluorophore Sytox Green^®^ was evaluated. This fluorophore has the capacity to bond to the nucleic acids of cells with alterations to the cytoplasmic membrane integrity, but not in intact cells [39]. The results of the quantitative determination of the fluorescence emission through flow cytometry are shown in Figure 3. The analysis of the results suggest that as the concentration of ΔM4 increases, there is a significant increase in the fluorescence of Sytox Green^®^, indicating that the peptide has a direct effect on the permeability of A375 melanoma and A431 carcinoma cell membranes. On the other hand, no significant differences are observed in the mean fluorescence intensity (MFI) of Sytox Green^®^ in HaCaT cells, which indicates that at concentrations of ΔM4 that are active against cancer cells, the integrity of non-tumoral cell membranes is not altered.

#### 3.2.3. Evaluation of the Interaction of ΔM4 with the Membrane Lipids

The wavenumber peak position of the ν_s_CH_2_ band is a recognized parameter associated with the bilayer order and packing of the lipid membranes. The lipid bilayer has different phases depending on temperature. At temperatures below the phase transition (gel phase), ν_s_CH_2_ lies around 2850 cm^−1^; while at temperatures above the phase transition (liquid crystalline phase), the band lies around 2853 cm^−1^. Based on the temperature dependence of the wavenumber of the methylene groups of the lipid system, it is possible to obtain the phase transition temperature (T_m_). This physicochemical parameter has a characteristic value for each phospholipid depending on the length of the acyl chains [40] and on the structure of the head groups [41].

**Figure 4:**
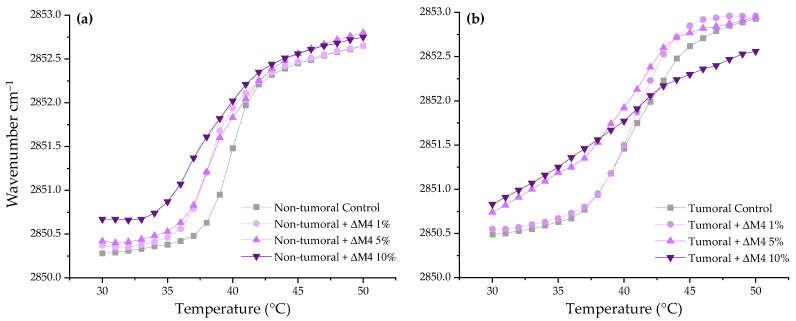
Methylene vibration bands as a function of temperature of (**a**) non-tumoral membrane representative model DPPC/SM/DPPE 4.35:4.35:1 (*w*/*w*) and (**b**) tumoral cell membrane model DPPC/SM/DPPE/DPPS/3.85:3.85:0.8:1.5 (*w*/*w*) in the presence of different concentrations of ΔM4.

However, in this study, two multicomponent lipid systems were selected in order to better represent the composition of the non-tumoral and tumoral cell membranes. Figure 4a represents wavenumber values of the peak positions of the DPPC/SM/DPPE 4.35:4.35:1 (*w*/*w*) acyl chains for the pure non-tumoral lipid system against the temperature, at different concentrations of ΔM4. In the absence of the peptide the non-tumoral lipid system has a T_m_ of 39.9 °C (Table 2). The interaction of ΔM4 results in an increase in the methylene peak positions, which is associated with a fluidization effect of the membrane. The highest effect is observed at 10 molar%, where the T_m_ shifts by 1.6 °C.

The results of the temperature dependence of the wavenumber values of the peak positions of the acyl chains for the pure tumoral lipid system at different concentrations of ΔM4 are represented in Figure 4b. The pure tumoral lipid system has a T_m_ of 41 °C, and the interaction of increasing peptide concentrations with the lipid system induces a fluidization of the acyl chains. At the highest concentration evaluated, of 10 molar%, the transition is almost lost, the T_m_ shifts by 2.4 °C, and the sigmoidal shape of the curve is altered as a consequence of the destabilizing effect induced by the peptide.

#### 3.2.4. Determination of the Secondary Structure of ΔM4

The mechanism of action of the membrane-active peptides is based on the hypothesis that when most peptides interact with the membrane environment they undergo a conformational change from random to α-helical structure. The conformational change has been proposed as a crucial step in the mechanism of membrane-active peptides. Based on this reason, the secondary structure of ΔM4 was predicted in buffer, and in the presence of three different representative lipid systems to follow the conformational change of the peptide. The obtained results are presented in Table 3.

The analysis of the prediction of the secondary structure of ΔM4 shows that the peptide has a random coil structure in buffer. However, when the peptide interacts with the non-tumoral system, there is a conformational change due to interaction with the lipid environment. For the tumoral system, the increase in the α-helix structure of the peptide is higher in comparison with that of the non-tumoral system. The last system evaluated is liposomes of pure phosphatidylserine, which are fully negatively charged on the surface of the liposomes. For this lipid system, the results show the higher conformational change of ΔM4 in comparison with the other two systems.

### 3.3. Exposure of Phosphatidylserine in Skin Cells after ΔM4 Treatment

**Figure 5:**
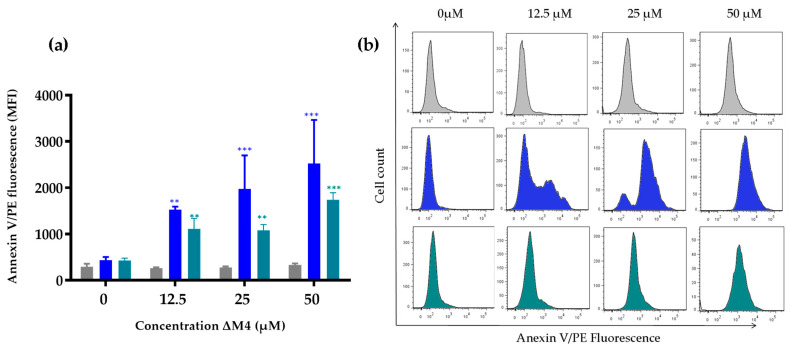
Exposure of PS after ΔM4 treatment. HaCaT (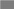), A375 (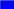), and A431 (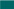) cells were treated with ΔM4 peptide for 24 h at different concentrations (12.5, 25, and 50 µM), and subsequently stained with annexin-PE and analyzed by flow cytometry. (**a**) The bar chart indicates an increase in fluorescence related to the expression of PS. (**b**) The flow cytometry histograms of annexin V/PE fluorescence intensities plotted against cell counts obtained from the analysis of untreated cells and cells treated with ΔM4 (12.5, 25, and 50 µM). Three independent experiments were conducted and results of one representative experiment are reported. Using one-way ANOVA, the differences were obtained with respect to non-treated cells, where **p ≤ 0.01, *** *p* ≤ 0.001.

Considering that annexin V presents an affinity for the charged lipid phosphatidylserine at the membrane, flow cytometry experiments were carried out in order to determine if different concentrations of the peptide ΔM4 on HaCaT, A375 melanoma, and A431 cells induce the exposure of this phospholipid, which is indicative of apoptosis. The obtained results are summarized in Figure 5a. The analysis suggests that the treatment with ΔM4 induces the translocation of the negatively charged phosphatidylserine from the inner to the outer leaflet of the A375 melanoma and A431 carcinoma cell membranes. A significant increase can be observed in the percentage of cells that are positive for annexin V in a dependent concentration effect, especially in A375 cells. On the other hand, the binding results for the HaCaT cells show no significant differences in the fluorescence of annexin V/PE with respect to the non-treated cells.

Figure 5b shows illustrative histograms obtained by flow cytometry and the quantification of the mean fluorescence intensity. The analysis of the results demonstrates the increase in phosphatidylserine exposure in A375 and A431 cells at all peptide concentrations evaluated, in comparison with the non-treated cells.

## 4. Discussion

Cases of skin cancer continue to rise around the world at an alarming rate. Despite programs of prevention and self-care, the search for and evaluation of potential new therapeutic agents for the treatment of skin cancer continues to be necessary. In the past decade, many studies have focused on the evaluation of peptides as potential anticancer agents, and the use of these is developing into a promising strategy for the treatment of many types of cancer, including melanoma [42,43,44]. This research was focused on evaluating the activity of the synthetic peptide ΔM4 against non-tumoral HaCaT epidermal cells, A375 melanoma-type, and A431 epidermoid carcinoma cells. The results obtained by the MTT assay demonstrate that ΔM4 significantly and selectively reduces the viability of melanoma and carcinoma cells in comparison with non-tumoral HaCaT epidermal cells. The IC_50_ values obtained from the viability curves show that ΔM4 is almost five times more active for A375 and A431 cells than for non-tumoral HaCaT cells. Using the IC_50_ values for the tumoral and non-tumoral cells, the SX was calculated. Based on the results, ΔM4 could be considered promising, given that a value over 100 is considered an initial sign of selectivity of the drug or substance evaluated [45]. The selectivity of bioactive peptides such as ΔM4 could be explained based on their physicochemical characteristics. This is a synthetic peptide composed of 20 amino acids, which is cationic (+7) at physiological pH and has a hydrophobicity of 50% [26]. The peptide’s charge is one of its most important characteristics, given that this is related to the first mechanism of action for bioactive peptides proposed by numerous authors. Most peptides with recognized biological activity present a positive charge ranging from +2 to +9 [46]. Peptide mechanism of action starts with the attraction between the positively charged amino acids of the peptide and the negatively charged groups of the cancer cell membranes [47,48]. This interaction could be explained by the composition of the membrane, which has a crucial role given that the tumor cells have differentiating characteristics such as the exposure of the negatively charged phospholipid phosphatidylserine towards the external side of the membrane, as well as the presence of proteoglycan chains in the form of heparan sulfate, which are mucins that contribute to the initial electrostatic interaction with the peptide [49,50].

Non-tumoral membranes are considered neutral due to the presence of zwitterionic phospholipids; moreover, the presence of cholesterol in the membrane modulates the fluidity and could inhibit the lytic effect of the peptides. Matsuzaki and co-workers found that the depletion of cholesterol in the erythrocytes leads to a greater sensitivity to the presence of magainin, which suggests that cholesterol could have a protective effect against active peptides at the membrane level [51]. In addition, it has been demonstrated that the membranes of cancer cells present higher fluidity in comparison with the non-tumoral cell membranes, and the presence of microvilli increases the area of exposure, thereby favoring interaction with the peptide [42]. Numerous studies have identified peptides with potential antitumoral activity, such as melittin and cecropin A, which have been shown to be active against several cancer lines. These peptides have even been used in a synergic manner with conventional chemotherapies, as an alternative that can reduce the peptide cytotoxic activity and have shown efficiency in the elimination of cancer cells [44,52].

Our research group previously reported a study on the effect of ΔM4 against a melanoma cell line at morphological level, considering the effects both on mitochondrial function and cell cycle progression [26]. However, considering that CAPs generally exhibit a mechanism that leads to the loss of the membrane integrity, this study included experiments focused on understanding the effect of ΔM4 on HaCaT, A375, and A431 cell membranes. In order to explore whether ΔM4 antitumoral activity could be related to a membrane effect, LDH experiments were performed. LDH is an enzyme contained in the cytoplasm that is released into the peripheral medium when the cell membrane is located [53]. The results suggest that ΔM4 induces a higher membrane effect against A375 and A431 cells in comparison with the HaCaT control cell line, which corroborates the selectivity observed in the viability studies. Concentrations above 12.5 µM could be related to a membrane-destabilizing mechanism induced by the peptide.

In order to confirm whether ΔM4 is able to induce a membrane-permeabilizing effect, the Sytox Green^®^ uptake evaluation was performed. The results show that, starting from 25 µM of the peptide, there is a significant increase in the fluorescence of the tumor cells, which is indicative that the cell membrane is compromised. It has been proposed that peptides have a critical concentration in exerting the mechanism of action. After the peptide is electrostatically bound to the membranes, the peptides are inserted into the membrane, destabilizing and altering the hydrophobic core. For this reason, infrared spectroscopy experiments were carried out with the aim of identifying the affinity of the peptide ΔM4 to lipids present in a model membrane representative of cancer cells. The results obtained demonstrate that the peptide ΔM4 has the capacity to induce a destabilization effect on the synthetic lipids representative of tumoral membranes in comparison with the non-tumoral model. This interaction modifies the phase transition temperature characteristic of the lipid system, and correlates with the results obtained for MTT, LDH, and Sytox, where ΔM4 is much more active against tumor cells than against non-tumoral cells.

Given that the most accepted mechanism of action of peptides involves a change in the secondary structure when the peptide interacts with cell membranes, infrared spectroscopy studies were carried out to monitor the conformational change of ΔM4 in different lipid systems by FT-IR. The results indicate that the peptide adopts an amphipathic helical structure in the presence of a lipid environment. In this sense, ΔM4 conformational change can be induced by the negatively charged lipid headgroups of membranes. These results correlate very well with our previous study where the helical wheel projection and the 3D structure of the ΔM4 show an polar/a polar structure, one rich in the hydrophobic residues and the other bearing a polar surface [26]. This polar face might be involved in the interaction of ΔM4 and the tumoral membrane of A375 and A431 membrane cells, inducing the membrane destabilizing mechanism.

Finally, flow cytometry experiments were carried out to determine whether treatment with ΔM4 is related to potential apoptotic mechanism at concentrations close to 25 µM. The process of cell death mediated by apoptosis is recognized by an alteration of the plasma membrane composition by the exposure of phosphatidylserine, while the membrane structure remains intact.

The exposure of PS can be followed by its affinity for annexin V, a recognized binding protein. It has been described that the exposure of phosphatidylserine from the internal face of the cellular membrane to the external face through the action of translocases is key for the recognition of apoptotic cells by macrophages [54,55]. The flow cytometry results show that the fluorescence of the annexin V/PE conjugate increases preferentially in the tumorigenic cell lines, in comparison with the HaCaT control cell line. Riedl et al. demonstrate that the peptide R-DIM-P-LF11-322 induces apoptosis in melanoma cells after one hour of treatment at 20 µM. The authors suggest that the peptide must enter the cell in order to reach the mitochondria and thereby cause cell death through the intrinsic route [56]. Similarly, other peptides such as TAT-Bim have been reported to have the ability to cross the cell membrane and induce apoptosis through the activation of caspase 3 in various types of cancer cells including melanoma, at concentrations between 2 and 10 µM [57]. Studies by Lewis et al. (2018) showed that the peptide nisin Z induced selective toxicity in A375 melanoma cells at concentrations above 100 µM, having a negative effect on cell energy metabolism, affecting glycolysis and mitochondrial respiration, increasing the generation of reactive oxygen species, and ultimately provoking cell death by apoptosis [58].

It has been reported that some peptides can have multiple mechanisms of action in inducing cell death, mainly associated with necrosis or apoptosis, and depending on the concentration used. Zhou et al. demonstrated that when lysine is substituted by leucine in the sequence of the peptide mauriporin, thereby modifying the lytic action of the peptide, an apoptotic effect is obtained on cervical cancer cells (HeLa) [59]. Another example of this double cell death mechanism was reported with the peptide LTX-315, which is also used as the control peptide in the present study. The first publications on the peptide LTX-315 defined it as an oncolytic agent that is active against various cancer cell lines including melanoma [36]. However, subsequent studies demonstrated its capacity to generate an immunological response through the expression of DAMPs in the treated cells [30,60]. Moreover, studies linked LTX-315 with the capacity to induce apoptosis through the mitochondrial route at concentrations of 3.5 µM, while at higher concentrations (17 µM), a necrotic effect via alteration of the plasmatic membrane was evidenced. This shows the capacity of this peptide to exert different cell death pathways depending on the concentration evaluated [30,61]. A final example is the study of Chang et al., who described the cytotoxic effect of hepcidin, a peptide obtained from tilapia fish (*Oreochromis mossambicus*) with the capacity to induce necrosis at concentrations above 100 µM, and apoptosis at lower concentrations (50 µM), in hepatocellular carcinoma and fibrosarcoma cells [62].

In summary, the results of this study suggest that ΔM4 exerts a destabilizing effect on A375 melanoma and A431 epidermoid carcinoma cell membranes, inducing cell death. This mode of action is related to peptide–lipid interactions that destabilize the membrane by inducing fluidity changes [63]. This mechanism is also supported by the flow cytometry experiments through Sytox uptake. However, considering that the results obtained with annexin suggest a potential pro-apoptotic effect, it is recommendable to carry out subsequent analyzes to confirm this cell death pathway.

## 5. Conclusions

Our study demonstrates the selective antitumoral activity of the peptide ΔM4 against A375 melanoma and A431 epidermoid carcinoma cells, with minimal effects on non-tumoral HaCaT cells. Additionally, our research explores the mechanism of action by demonstrating that the peptide is able to interact with the membrane lipids, inducing changes in membrane fluidity and subsequent destabilization of the bilayer core.

The concentration-dependent membrane effect of ΔM4 indicates a potential relation to alterations in cell permeability, which may provide an explanation for the observed surface exposure of PS in cancer cells. Notably, PS exposure is a crucial event linked to early apoptosis. Thus, our study proposes that treatment with ΔM4 has the potential to induce apoptosis in cancer cell lines, highlighting its promising prospects for future research. The results contribute to the growing evidence supporting the development of ΔM4 as a promising candidate for targeted skin cancer therapies. Nonetheless, it is essential to acknowledge that further studies are necessary to understand the precise mechanism of action employed by ΔM4 in skin cancer cells. Future research efforts should focus on elucidating the specific molecular interactions between the peptide and cancer cell membranes, as well as investigating downstream signalling pathways and the subsequent cellular responses.

## Figures and Tables

**Table 1:** Half-maximal inhibitory concentration (IC_50_) obtained for the peptides ΔM4 and LTX-315 on non-tumoral HaCaT, A375 melanoma, and A431 epidermoid carcinoma cells. Selectivity index (SX) was calculated for A375 melanoma, and A431 epidermoid carcinoma cells.

Cell Line	Concentration (µM)		Selectivity Index (SX) *	
	ΔM4	LTX-315	ΔM4	LTX-315
HaCaT	98	40	---	---
A375	20	65	490	61
A431	26	65	377	61

* SX value > 100 represents that cytotoxic effect is more selective in cancer cells.

**Table 2:** Effect of ΔM4 on the main transition temperature (T_m_) of the non-tumoral and tumoral lipid systems. Standard deviations are ≤0.1 °C.

ΔM4 Concentration (molar%)	Phase Transition Temperature (°C)	
	Non-Tumoral	Tumoral
0	39.9	41.0
1	38.7	40.7
5	39.2	39.6
10	38.3	38.6

**Table 3:** Prediction of the secondary structure of ΔM4 in HEPES buffer, non-tumoral, tumoral, and phosphatidylserine model membranes at 37 °C. The calibration method used for the prediction of the secondary structure has a deviation of ±4.4% for the helix.

	System	α-Helix Prediction (%)
ΔM4 +	Hepes	3.2
	Non-tumoral	49.5
	Tumoral	61.0
	POPS *	70.3

* POPS liposomes were prepared in Hepes buffer 20 mM, NaCl 500 mM, and 1 mM EDTA.

## Data Availability

The data involved in this paper have been presented in articles and supporting materials in the form of diagrams or tables.
